# LC-MS/MS Tandem Mass Spectrometry for Analysis of Phenolic Compounds and Pentacyclic Triterpenes in Antifungal Extracts of *Terminalia brownii* (Fresen)

**DOI:** 10.3390/antibiotics6040037

**Published:** 2017-12-13

**Authors:** Enass Y. A. Salih, Pia Fyhrquist, Ashraf M. A. Abdalla, Abdelazim Y. Abdelgadir, Markku Kanninen, Marketta Sipi, Olavi Luukkanen, Mustafa K. M. Fahmi, Mai H. Elamin, Hiba A. Ali

**Affiliations:** 1 Department of Forest Products and Industries, Faculty of Forestry, PO Box 13314, University of Khartoum, Khartoum 11111, Sudan; amahmed@uofk.edu (A.M.A.A.); ayabdelgadir@uofk.edu (A.Y.A.); mkfahmi@uofk.edu (M.K.M.F.); 2 Commission for Biotechnology and Genetic Engineering, PO Box 2404, National Centre for Research, Khartoum, Sudan; hibaali@hotmail.com; 3 Faculty of Pharmacy, Division of Pharmaceutical Biosciences, PO Box 56, University of Helsinki, FIN-00014 Helsinki, Finland; pia.fyhrquist@helsinki.fi; 4 Viikki Tropical Resources Institute (VITRI), Department of Forest Sciences, PO Box 27, University of Helsinki, FIN-00014 Helsinki, Finland; markku.kanninen@helsinki.fi (M.K.); Marketta.Sipi@helsinki.fi (M.S.); olavi.luukkanen@helsinki.fi (O.L.); 5 Department of Phytochemistry, Faculty of Pharmacy, PO Box 477, University of Sciences and Technology, Omdurman, Sudan; maielamin15@gmail.com

**Keywords:** Africa, *Terminalia brownii*, antifungal stem wood and bark extracts, *Aspergillus*, *Nattrassia*, Fusarium, LC-MS/MS, flavonoids, ellagitannins, stilbenes, triterpenes

## Abstract

Decoctions and macerations of the stem bark and wood of *Terminalia brownii* Fresen. are used in traditional medicine for fungal infections and as fungicides on field crops and in traditional granaries in Sudan. In addition, *T. brownii* water extracts are commonly used as sprays for protecting wooden houses and furniture. Therefore, using agar disc diffusion and macrodilution methods, eight extracts of various polarities from the stem wood and bark were screened for their growth-inhibitory effects against filamentous fungi commonly causing fruit, vegetable, grain and wood decay, as well as infections in the immunocompromised host. Ethyl acetate extracts of the stem wood and bark gave the best antifungal activities, with MIC values of 250 µg/mL against *Nattrassia mangiferae* and *Fusarium verticillioides*, and 500 µg/mL against *Aspergillus niger* and *Aspergillus flavus*. Aqueous extracts gave almost as potent effects as the ethyl acetate extracts against the *Aspergillus* and *Fusarium* strains, and were slightly more active than the ethyl acetate extracts against *Nattrassia* *mangiferae*. Thin layer chromatography, RP-HPLC-DAD and tandem mass spectrometry (LC-MS/MS), were employed to identify the chemical constituents in the ethyl acetate fractions of the stem bark and wood. The stem bark and wood were found to have a similar qualitative composition of polyphenols and triterpenoids, but differed quantitatively from each other. The stilbene derivatives, *cis-* (**3**) and *trans*- resveratrol-3-*O*-β-galloylglucoside (**4**), were identified for the first time in *T. brownii*. Moreover, methyl-(*S*)-flavogallonate (**5**), quercetin-7-β-*O*-di-glucoside (**8**), quercetin-7-*O*-galloyl-glucoside (**10**), naringenin-4′-methoxy-7-pyranoside (**7**), 5,6-dihydroxy-3′,4′,7-tri-methoxy flavone (**12**), gallagic acid dilactone (terminalin) (**6**), a corilagin derivative (**9**) and two oleanane type triterpenoids (**1**) and (**2**) were characterized. The flavonoids, a corilagin derivative and terminalin, have not been identified before in *T. brownii*. We reported earlier on the occurrence of methyl-*S*-flavogallonate and its isomer in the roots of *T. brownii*, but this is the first report on their occurrence in the stem wood as well. Our results justify the traditional uses of macerations and decoctions of *T. brownii* stem wood and bark for crop and wood protection and demonstrate that standardized extracts could have uses for the eco-friendly control of plant pathogenic fungi in African agroforestry systems. Likewise, our results justify the traditional uses of these preparations for the treatment of skin infections caused by filamentous fungi.

## 1. Introduction

Fungal contamination is both a pre- and a post-harvesting problem in crop production and poses a continuous and growing threat to global food crop production [1,2]. Some of the fungal species generally considered to be phytopathogens, such as *Aspergillus* spp., are also known to be increasingly significant as human pathogens, especially in the immunocompromised host [3,4,5].

*Aspergillus niger* (van Tieghem, 1867) and *Aspergillus flavus* (Link, 1809) are both human [6,7] and plant pathogens [8]. As human pathogens, especially *A. flavus*, but also *A. niger* cause aspergillosis in immunocompromised individuals [9,10]. *A. flavus* causes grain crop infections in maize (*Zea mays* L.), leading to a substantial decrease in the commercial value of maize crop due to aflatoxin contamination [11]. Moreover, *A. flavus* is often the causative agent of wood decay in timber and houses [12]. *Nattrassia mangiferae* [(Syd. and P. Syd.) B. Sutton and Dyko], previously known as *Hendersonula toruloi* Nattrass (HT) and *Dothiorella mangiferae* (Syd. and P. Syd), is a wound-invading dematiaceous (brown-pigmented) phytopathogenic fungus infecting hard wood species of *Citrus, Mangifera* and *Eucalyptus* and soft wood coniferous subtropical and tropical trees, causing dieback and vascular wilt diseases [13,14]. *Nattrassia mangiferae* is also a human pathogenic fungus, especially in immunocompromised individuals [15], and is even known to cause community acquired infections in rural farmer societies worldwide [13]. *Fusarium verticilloides* and some other *Fusarium* species infect maize ears (husks) causing maize ear rot disease and contaminate maize grains with fumonisin mycotoxins leading to major pre- and post-harvest losses [16]. *Fusarium* spp. mycotoxins are toxic [17,18], and fumonisin has been found to cause cancer in mammalians [19]. Another species of *Fusarium*, *F. oxysporum* is the causative agent of the “Panama disease” affecting the banana (*Musa paradisiaca*), the staple food of a large part of Africa.

Currently used fungicides are costly and toxic to the environment [20,21]. Besides, phytopathogenic and human pathogenic filamentous fungi have developed resistance to many conventional fungicides and to antibiotics [22,23,24,25,26]. Thus, new effective, less toxic, affordable and readily available antifungals are needed [26]. Tropical and subtropical plants are known to contain a wide range of defense compounds due to their needs for constant production of defense compounds throughout the year as well as due to the high biodiversity in rain forests, woodlands and savannahs [27]. Thus, tropical plant species used for fungal infections in African traditional medicine as well as for protection of crop plants against fungal contamination, are expected to be good sources for new antifungal compounds [28,29].

The pantropical genus of *Terminalia* (Combretaceae) contains a number of species known for their antifungal effects. Antifungal activities against *Aspergillus niger* and *A. flavus* have been reported for the Asian species, *Terminalia alata*, *T. arjuna*, *T. bellerica, T. catappa* and *T. chebula* [30,31]. Approximately 30 species of *Terminalia* occur in Africa [32]. However, despite of their frequent uses in traditional medicine for treatment of fungal infections in humans and in traditional agriculture for prevention of fungal crop plant contamination, only a small portion of these species have been studied in depth for their antifungal activity and/or antifungal compounds. Among those African *Terminalia* species investigated for their antifungal potential, either against yeasts or filamentous fungi or both, are *T. avicennoides*, *T. spinosa*, *T. sericea* and *T. nigrovenulosa* [33,34,35,36]. Ellagitannins, ellagic acid derivatives, stilbenes, lignans, flavonoids and pentacyclic triterpenes were reported from some African species of *Terminalia*, such as *T. horrida*, *T. sericea, T. superba* and *T. macroptera* [33,34,37,38,39,40,41,42,43,44,45]. Most of these phytochemical investigations did not include antifungal screening of the characterized compounds, however.

**Figure 1:**
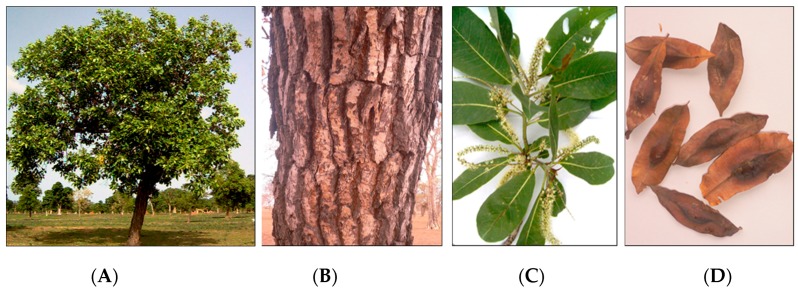
*Terminalia brownii*. (**A**) tree in savannah woodland; (**B**) stem bark; (**C**) flowers and leaves; (**D**) fruits. Photo: E. Y. A. Salih and Dr. H. H. Gibreel, 2006.

*Terminalia brownii* (Fresen.) is a deciduous tree distributed throughout East African savannah regions in a wide range of temperature, rain fall and soil conditions (Figure 1) [46]. In Sudan, *T. brownii* occurs in low and high rainfall zones in Blue Nile state and El-Gadarif in the eastern part of the country and Kordofan and Darfour states in the western part of Sudan. In Sudan, *T. brownii* grows in natural forested areas such as savannah woodlands where it is listed as an endangered species due to overexploitation [47]. *T brownii* has been found to be exceptionally resistant against various pathogenic fungi that affect crops, and is frequently used in traditional agroforestry for crop plant protection [21]. Similarly to many other plant species, such as *Melianthus comosus* [48], also decoctions of various parts of *T. brownii* have traditional applications as fungicides against fungal contamination in harvested crop plants.

*Terminalia brownii* has been found to be a rich source of oleanane- and ursane-type pentacyclic triterpenoids, such as arjunic acid, galloyl arjunic acid, tomentosic acid, sericic acid, arjungenin, sericoside, betulinic acid, monogynol A and arjunglucoside [21,49]. In addition, a new oleanane type triterpenoid, designated as 3β,24-*O*-ethylidenyl-2α,19α-dihydroxyolean-12-en-28-oic acid, was identified in an ethyl acetate extract of the stem bark of *T. brownii* [49]. Moreover, β-sitosterol and ellagic acid derivatives have been characterized from the leaves and stem bark of *T. brownii* [21,49]. In addition, a number of unknown and known ellagitannins, including methyl-(*S*)-flavogallonate and its derivative as well as α,β-punicalagin and α,β-terchebulin have been described form the roots and the stem bark, respectively [50,51]. Moreover, a chromone derivative designated as terminalianone has been found in the stem bark of *T. brownii* [52]. However, to our knowledge, the antifungal effects of the mentioned compounds were not investigated, with the exception of arjungenin, β-sitosterol and betulinic acid [21].

Although *T. brownii* extracts are used traditionally against fungal phytopathogens and to treat human fungal infections in Sudan and in other countries of Africa, there are a limited number of reports on their in vitro antifungal activity against filamentous fungi affecting crop production and human health. Moreover, only a small number of antifungal compounds in *T. brownii* have been characterized to date [21,49], and to the best of our knowledge, no flavonoid structures have been studied in this species of *Terminalia*. Therefore, the current study was performed to verify the antifungal effects of decoctions and macerations, reported to be used for fungal infections in traditional medicine and as fungicides in traditional agriculture. In addition, extracts of various polarities made from the stem bark and wood of *T. brownii* were tested for their growth-inhibitory effects. For the screenings, significant phytopathogenic and human pathogenic opportunistic fungi of the genera *Aspergillus*, *Nattrassia* and *Fusarium* were used. Thin layer chromatography (TLC) and RP-HPLC/DAD were used to study the phytochemical composition of the ethyl acetate extracts of the stem wood and bark. Tandem mass spectrometry (LC-MS/MS) was used to elucidate the molecular masses of flavonoids, triterpenes, ellagitannins and stilbenes in an antifungal stem wood extract of *T. brownii*.

## 2. Results

### 2.1. Antifungal Effects of Extracts of Terminalia brownii Stem Bark and Wood

The results of the growth inhibition of various extracts of *Terminalia brownii* stem bark and wood against *Aspergillus*, *Nattrassia* and *Fusarium* strains are shown in Table 1. When compared to the other extracts, the ethyl acetate extracts of the stem wood and bark gave the highest antifungal activity. This result is in accordance with other authors, who also reported that especially ethyl acetate extracts of the stem bark of *T. brownii* give good antifungal effects against sweet potato infecting fungi, such as *Aspergillus niger* and *Fusarium solanii* [21]. The reference antifungal used in our tests, amphotericin-B, was more growth-inhibitory than the ethyl acetate extracts, however, although the differences in potency between amphotericin-B and the ethyl acetate extracts were not big, considering that we used plant extracts instead of pure compounds present in these extracts (Table 1).

Our results demonstrate that *Nattrassia mangiferae* and *Fusarium verticilliodies* were especially sensitive to the ethyl acetate extracts of *T. brownii* stem wood and bark, giving MIC values of 250 µg/mL, whereas *Aspergillus niger* and *A. flavus* were more resistant to these extracts, demonstrating MIC values of 500 µg/mL (Table 1). We found that the obtained MIC values correlated well with the sizes of the inhibition zones produced by these ethyl acetate extracts, so that small MIC values were coupled to large diameters of the inhibition zones (Table 1). To the best of our knowledge, this is the first report on antifungal effects of *T. brownii* against *Nattrassia mangiferae*.

**Figure 2:**
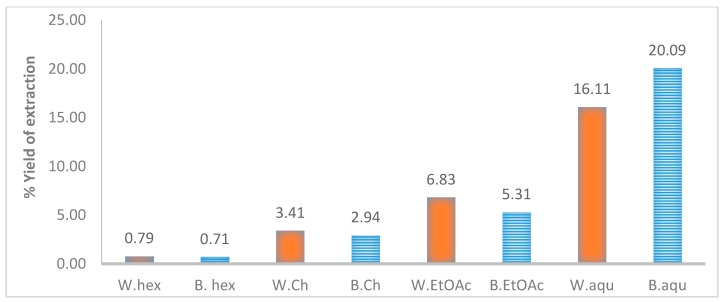
Percentage yield (% *w*/*w*) resulting from sequential extraction and liquid/liquid partition of the stem wood and stem bark of *Terminalia brownii*. W, stem wood; B, stem bark; hex, hexane extract; Ch, choloroform extract; EtOAc, ethyl acetate extract; aqu, aqueous extract.

Interestingly, we also found that aqueous extracts of the stem bark and wood of *T. brownii* gave good growth-inhibitory effects (Table 1). Compared to the other extracts these aqueous extracts gave especially high extraction yields of 20 and 16%, respectively for bark and wood (Figure 2). Thus, our results justify the traditional application of macerations (water extracts) of the stem wood and barks of *T. brownii* for the preservation of grains and wooden house poles and for traditional medicinal treatment of fungal infections caused by *Aspergillus*, *Nattrassia* and *Fusarium*. Earlier studies have indicated that aqueous stem bark extracts of *T. brownii* are growth-inhibitory also against yeast species, such as *Candida albicans* and *Cryprococcus neoformans* and in addition the aqueous extracts were found to be less toxic than other extracts against brine shrimps [53]. Thus, standardized aqueous extracts of *T. brownii* stem wood and bark could be used to treat fungal infections and fungal contamination.

When compared to the more polar water and ethyl acetate extracts, we found that chloroform extracts of the stem wood and bark of *T. brownii* were slightly antifungal, while the petroleum ether extracts were devoid of antifungal activity (Table 1). In contrast to our results, in an earlier investigation, it was found that an n-hexane extract of the stem bark of *Terminalia brownii* was active against another *Aspergillus* species, *A. fumigatus* [54]. Perhaps this result might indicate that different species of *Aspergillus* differ to their sensitivity to non-polar extracts of *T. brownii*, so that *A. fumigatus* is more sensitive than *A. niger* and *A. flavus*.

### 2.2. Results from the Phytochemical Screening of Antifungal Ethyl Acetate Extracts of T. brownii Stem Wood and Bark

Owing to our promising antifungal results for the ethyl acetate extracts of the stem bark and wood of *T. brownii*, and to the few existing earlier records on the activity of this species against filamentous fungi, we investigated the secondary compound composition and molecular masses as well as the fragmentation patterns of phenolic compounds and triterpenoids of these extracts.

#### 2.2.1. TLC Results

RP-18 thin layer chromatograms of the ethyl acetate extracts of the stem wood and stem bark of *T. brownii* gave a negative reaction with Dragendorff reagent, suggesting that these extracts were devoid of alkaloids. Pink to purple colors were developed upon spraying with vanillin-H_2_SO_4_, which suggested the presence of triterpenoid and phenolic compounds. Spraying the TLC plates with aluminum trichloride (AlCl_3_) and Natural Product reagent (NPR), revealed the presence of flavonoids, since color changes from quenching fluorescence to yellow, orange or blue color, typical for flavonoidal acids or other phenolic acids, could be observed at 366 nm [57].

#### 2.2.2. HPLC-UV/DAD Results

**Figure 3:**
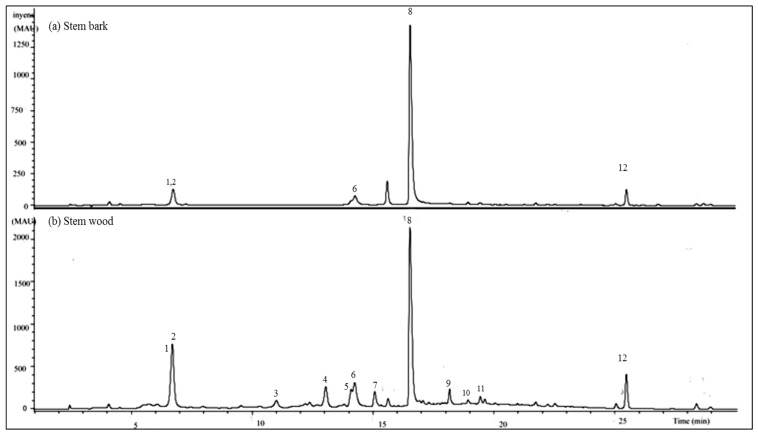
RP-HPLC/DAD chromatograms of ethyl acetate extracts of *T.brownii*. (**A**) stem bark and (**B**) stem wood extracts at 254 nm. (**1**) and (**2**) Oleanane type triterpenoids; (**3**) cis-resveratrol-3-*O*-β-galloyl-glucoside; (**4**) trans-resveratrol-3-*O*-β-galloyl-glucoside; (**5**) Methyl-(*S*)-flavogallonate; (**6**) Gallagic acid dilactone (Terminalin); (**7)** Naringenin-4′-methoxy-7-pyranoside; (**8**) Quercetin-7-ß-*O*-diglucoside; (**9**) Corilagin derivative; (**10**) Quercetin-7-*O*-galloyl-glucoside; (**11**) unknown ellagitannin; (**12**) 5,6-dihydroxy-3′,4′,7-trimethoxy flavone.

HPLC-UV/DAD fingerprints of the ethyl acetate extracts of the stem bark and stem wood of *Terminalia brownii* are presented in Figure 3. Altogether twelve compounds with retention times between 6.8 and 25.5 min could be identified using internal standards and a computer library for standard compounds. At the wavelengths of 320 and 254 nm, which were used for detection of stilbenes and flavonoids, the wood ethyl acetate extract displayed a higher diversity of flavonoidal and stilbenoid compounds. For example, the *cis*- and *trans*-isomers of resveratrol 3-*O*-β-galloyl-glucoside (**3** and **4**) at Rt 11.1 and 13.2 min, respectively, as well as naringenin-4′-methoxy-7-pyranoside (**7)** at 15.3 min, the corilagin derivative (**9)** at Rt 18.2 min, and quercetin-7-*O*-galloyl glucoside (**10)** at Rt 18.4 min, were present in the wood extract but absent from the stem bark extract as shown in Figure 3.

Because of the high number of compounds present in the wood ethyl acetate extract and due to this extract being slightly more antifungal than the stem bark, at least in terms of the sizes of the diameters of inhibition zones (Table 1), this extract was subjected to LC-MS/MS advanced analysis for identification of the major compounds.

#### 2.2.3. LC-MS/MS Results 

MS/MS combined with collision-induced dissociation (CID), has been found to enable the accurate identification of stilbenes and flavonoids in complex extracts with co-eluting peaks [58]. Therefore MS/MS was employed as the method of choice for the identification of compounds in an ethyl acetate extract of *T. brownii* stem wood. A total of twelve compounds were characterized by comparing the obtained molecular (precursor) ions and fragmentation patterns (i.e., product ions) from our LC-MS/MS data with data from the literature and with a computer library for the standard compounds (Table 2). The MS^2^, MS^3^ and MS^4^ ion chromatograms are presented in the supplemental part of this paper (Supplement 1).

**Figure 4:**
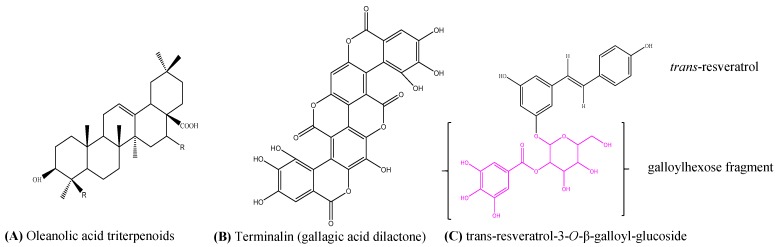
Chemical structures of some of the characterized compounds in the stem wood and stem bark of *T. brownii*. (**A**) oleanane type triterpenoids (compounds **1** and **2**); (**B**) Terminalin (compound **6**) and (**C**) *trans*-resveratrol-3-*O*-β-galloyl-glucoside (compound **4**).

We found that the stem wood of *T. brownii* contains two oleanane triterpenoid acids that co-eluted at 6.8 min (Figure 3 and Figure 4). For compound (**1**) a [M-H]^−^ molecular ion at *m*/*z* 469 was detected, whereas compound (**2**) gave a molecular ion of *m*/*z* 491. In the MS^2^ chromatograms, a fragment ion at *m*/*z* 425 was detected for compound (**1**) and at *m*/*z* 447 for compound (**2**) (Table 2, Supplement 1, Slide 1). These fragment ions indicate the loss of a carboxylic acid (-COOH) group ([M-H]^−^ for -COOH = 44) from both molecular ions. In agreement with our results, the loss of carboxylic acid at position 17 in pentacyclic triterpenoids was observed when using atmospheric pressure chemical ionization (APCI)-MS [59,60]. Moreover, we observed a fragment ion at *m*/*z* 407, which indicated the loss of H_2_O from *m*/*z* 425 in compound **1**. This kind of mass spectral fragmentation pattern is typical for oleanane type triterpenes [61,62], therefore confirming that compounds 1 and 2 are oleanane type triterpenes (Figure 4A).

In our HPLC-DAD system, compounds (**3**) and (**4**) eluted at Rt 11.1 and 13.2 min, respectively (Figure 3b). Both compounds showed an identical [M-H]^−^ molecular ion at *m*/*z* 541. Moreover, when subjected to MS^3^, both compounds provided fragment ions of *m*/*z* 227 and 314 (Table 2, Supplement 1, Slide 3 and 4). The fragment ion at *m*/*z* 314 indicates the presence of a galloylhexose fragment [63]. A comparison with the literature showed that the fragment ion at *m*/*z* 227 corresponds to the resveratrol unit [44]. Therefore, compounds (**3**) and (**4**) were tentatively assigned as resveratrol-3-*O*-β-galloyl-glucoside, respectively. Due to different retention times, the compounds were proposed to be *cis*- (**3**) and *trans*- (**4**) isomers of resveratrol-3-*O*-β-galloylglucoside (Figure 4C).

When subjected to MS^2^, compound (**5**) at Rt 14.1 min in HPLC-DAD (Figure 3b), gave an [M-H]^−^ molecular ion at *m*/*z* 483 (Table 2, Supplement 1, Slide 5). The loss of the two oxygen molecules {483 − 451 = 32} at MS^3^, gave a fragmentation ion at *m*/*z* 433. Also, the MS^3^ spectrum was devoid of the fragment of [M-H-CO_2_]^−^, which corresponds to a methyl ester molecule [64]. Therefore, and according to previous investigations [45,50], compound (**5**) was tentatively assigned the structure of methyl-(*S*)-flavogallonate.

Compound (**6**) at Rt 14.4 min (Figure 3b), gave a [M-H]^−^ molecular ion at *m*/*z* 601 (Figure 3b, Table 2). When subjected to MS^3^, compound (**6**) yielded the fragment ions at *m*/*z* 271 and 301, the later corresponding to free ellagic acid (Supplement 1, Slide 6). A molecular ion of *m*/*z* 601 and fragment ions at *m*/*z* 271 and 301 have been reported for gallagic acid [45]. Gallagic acid dilactone (syn. terminalin) has been reported in another species of *Terminalia, T. oblongata* [65]. Accordingly, compound (**6**) was tentatively assigned the structure of gallagic acid dilactone (terminalin) (Figure 4B). 

Compound (**7**) at Rt 15.3 min (Figure 3b) gave an [M-H]^−^ molecular ion at *m*/*z* 433 (Table 2). The deprotonation of [M-H]^−^ at MS^2^ resulted in a fragment ion at *m*/*z* 300, indicating the loss of one molecule of a pentose sugar ([M-H]-132) [63,66]. Moreover, MS^3^ of this compound yielded the loss of an Y0 fragment at *m*/*z* 271, corresponding to the cleavage of the aglycone fragment ion of the flavanone naringenin [66,67,68]. In the MS^3^, the [M-H]^−^ yielded a fragment at *m*/*z* 284, therefore indicating that the methoxy group occurs at position 4′ [66] (Supplement 1, Slide 7). As flavonoids commonly occur as *O*-glycosides and *O*-glycosylation occurs at position 7 in flavanones [63,69], compound (**7**) was tentatively assigned to naringenin-4′-methoxy-7-pyranoside.

The main compound (**8**) in the HPLC chromatogram at Rt 16.8 min (Figure 3) gave a [M-H]^−^ molecular ion at *m*/*z* 625 (Table 2). The main fragmentation product ion at *m*/*z* 301 in the MS^2^ and MS^3^ chromatograms indicated the loss of two glucose molecules ([M-H]^−^-2 × 162 Da) as well the presence of a quercetin aglycone moiety corresponding to *m*/*z* 301 [68] (Supplement 1, Slide 8). Since glucose is usually β-glycosidically linked to the flavonoid aglycone and *O*-glycosidic linking is usually occurring at position 7 on the A ring of flavonoids [69], compound (**8**) was tentatively identified as quercetin-7-β-*O*-diglucoside. 

Compound (**9**) at Rt 18.2 min (Figure 3b), gave a [M-H]^−^ molecular ion at *m*/*z* 633 (Table 2). MS/MS fragmentation resulted in a loss of a fragment product ion at *m*/*z* 481, corresponding to [M-galloyl-gallic acid. Other fragment product ions resulting from MS^2^ were; 463 [M-gallic acid]^−^, 300 (hexahydroxydiphenoyl-H) and 169 corresponding to gallic acid [45,70,71] (Supplement 1, Slide 9). Consequently, compound (**9**) is suggested to be a derivative of corilagin.

Compound (**10**) at Rt 18.4 min (Figure 3b) gave a [M-H]^−^ molecular ion at *m*/*z* 585 (Figure 3, Table 2). MS^2^ fragmentation of this compound resulted in the loss of a pyranose sugar corresponding to the fragment ion at *m*/*z* 132 and a galloyl unit corresponding to a fragment ion of *m*/*z* 153. Moreover, a fragment product ion at *m*/*z* 301 {[M-H]^−^-132-153} (Table 2), corresponding to the aglycone of quercetin, was present in the MS^2^ chromatogram [68] (Supplement 1, Slide 10). Consequently, compound (**10**) was tentatively assigned to be quercetin 7-*O*-galloyl-glucoside.

A polyphenol (Compound **11**) with a retention time of 19.1 min in HPLC-DAD (Figure 3b) gave a molecular ion at *m*/*z* 725. In the MS^2^ spectrum, the fragment ions of a hexose sugar were observed at *m*/*z* 665 and 503, indicating that the cleavage within this hexose sugar ring occurred at _0.3_X [M-H-61]^−^ [63,69]. Moreover, the loss of two oxygen molecules was noticed at MS^3^ [M-H-2 × 16]^−^. In the spectra at MS^4^, two fragments at *m*/*z* 391 and 379 were observed. These fragments resulted from the loss of one molecule of water and two methyl groups, respectively, from the fragment at *m*/*z* 409 in spectra MS^3^ ([M-H]^−^-H_2_O-2CH_3_]^−^ (Supplement 1, Slide 11). Thus, compound (**11**) is suggested to be identical to an unknown ellagitannin that we have reported earlier to occur in *T. brownii* roots [50].

Compound (**12**) at Rt 25.5 min (Figure 3b), gave a [M-H]^−^ molecular ion at *m*/*z* 343 (Table 2). MS^3^ and MS^4^ fragmentation of this compound resulted in the loss of three methyl groups (-CH_3_) corresponding to product fragment ions {[M-H]^−^ 343-328-313} (Table 2, Supplement 1, Slide 12). Moreover, in the fragment ion chromatogram resulting from MS^3^, a high intensity of the product fragment ion at *m*/*z* 313 could be observed indicating the loss of a methyl group ([M-H]^−^-15) (Table 2, Supplement 1, Slide 12). From this data, compound (**12**) was tentatively assigned as 5,6-dihydroxy-3′,4′,7-trimethoxyflavone.

## 3. Discussion

Pentacyclic triterpene saponins are known to complex with ergosterol and cholesterol in the fungal cell membrane, thus leading to loss of membrane integrity [72] and it has been found that triterpenoids decrease mycelial growth [73]. Accordingly, the triterpenes betulinic acid and arjungenin, isolated from ethyl acetate extracts of *T. brownii* stem bark were found to give good antifungal effects against *Aspergillus niger*, *Fusarium solanii* and *Fusarium oxysporum* with MIC values ranging from 50 to 200 µg/mL [21]. Therefore, we suggest that the two unknown oleanane-type triterpenes (**1**) and (**2**) would contribute significantly to the antifungal effects we have found for the ethyl acetate extract of the stem wood of *T. brownii* (Table 2, Figure 3).

In the genus *Terminalia*, resveratrol and its glucoside and rutinoside derivatives have been reported in *Terminalia prunioides*, *T. sericea* and *T. ferdinandiana* [44,74,75]. We reported here for the first time on the occurrence of the resveratrol derivatives, *cis-* (**3**) and *trans*-resveratrol-3-*O*-β-galloylglucoside (**4**) in *Terminalia brownii* stem wood. Besides, galloylglucoside derivatives of resveratrol have not been reported before in the genus *Terminalia*. Resveratrol and its derivatives are antifungal phytoalexins, protecting plants from pathogenic fungal and bacterial intrusion [76,77]. Several investigations on in vitro antifungal activities of resveratrol and its derivatives indicate good antifungal potential of this compound class [78,79]. Therefore, the good antifungal activity in the ethyl acetate extracts of *T. brownii* could partly be due to the resveratrol-galloylglucoside derivatives (**2**) and (**3**). To the best of our knowledge resveratrol-3-*O*-β-galloylglucoside has not been studied for its antifungal effects, which warrants further studies in this respect.

We reported here for the first time, on the occurrence of another ellagic acid derivative, gallagic acid dilactone (**6**)**,** in the stem wood of *T. brownii*. Gallagic acid, is an analogue to ellagic acid, containing four gallic acid residues [80] and has restricted occurrence in plants. Gallagic acid and its derivatives have been found in various parts of some other *Terminalia* species such as in the leaves of *T. catappa* and *T. oblongata* [65,81] and in the fruits of *Terminalia bellerica*, *Terminalia horrida* and *T. chebula* [45]. Gallagic acid is the fully lactonized form of the gallagyl moiety in the ellagitannin punicalagin, which is common in *Terminalia* spp. [45,82]. Gallagic acid has been found to give concentration-dependent growth-inhibitory effects against *Fusarium* and *Alternaria* [83]. Thus, it is possible that gallagic acid dilactone (**6**), which we found to be present in an ethyl acetate extract of the stem wood of *T. brownii*, could be an important contributor to the antifungal effects of this extract.

Ellagitannins have been found to inhibit the growth of *Fusarium* and *Alternaria* dose-dependently [83]. Even though the genus *Terminalia* is renowned to be especially rich in ellagitannins [45] only a few studies have been performed on the antifungal effects of ellagitannins isolated from *Terminalia* species. Some of the few investigations demonstrate that ellagitannins from *Terminalia* spp. could be valuable antifungal compounds. For example, punicalagin from the leaf of *T. brachystemma* was found to give a low MIC value of 6.25 μg/mL against *Candida* strains [61]. It was found, however, that some ellagitannins were not active against filamentous fungi, although activity was demonstrated against *Candida* and *Cryptococcus neoformans* [84]. Thus, it remains to be investigated whether the ellagitannins we have found in *T. brownii* stem wood, such as methyl-*(S)*-flavogallonate (**5**), the unknown ellagitannin (**11**) and the corilagin derivative (**9**) give low MIC values against filamentous fungi such as *Aspergillus, Nattrassia* and *Fusarium* spp. among others.

Our research resulted in the characterization of the flavonoids naringenin-4′-methoxy-7-pyranoside (**7**), quercetin-7-β-*O*-diglucoside (**8**), quercetin-7-*O*-galloylglycoside (**10**) and 5,6-dihydroxy-3,4,7-trimethoxy flavone (**12**) in the ethyl acetate extracts of stem wood and bark of *Terminalia brownii*. To the best of our knowledge this is the first time these flavonoids are reported to occur in *T. brownii*. We suggest that quercetin-7-β-*O*-diglucoside (**8**), which was quantitatively the main peak in both stem bark and wood extracts of *T. brownii*, contributes significantly to the antifungal effects of these extracts. Accordingly, several authors have reported that quercetin and its derivatives give good antifungal effects against *Aspergillus* and *Fusarium* strains [85,86,87] and for quercetin as low MIC values as 15 µg/mL were recorded against *Aspergillus niger*, *Fusarium moniliforme* and *F. sporotrichum* [88]. Furthermore, dihydroquercetin from barley suppressed the growth of *Fusarium* spp. [89]. However, it has been demonstrated that quercetin-glycoside was not as antifungal as its aglycone [90]. In contrast to quercetin, some other flavonoids have demonstrated strong antifungal effects as glycosides. For example, naringenin pyranoside demonstrated some antifungal activity with MIC values of 1600–3200 µg/mL against *Candida albicans* and *C. krusei* [91]. Therefore, naringenin-4′-methoxy-7-pyranoside, which we have found in the stem wood of *T. brownii*, is suggested to give some antifungal activity. Moreover, it has been found that flavonoids possessing methoxy groups are especially antifungal [87]. This would apply to naringenin-4′-methoxy-7-pyranoside (**7**) and 5,6-dihydroxy-3,4,7-trimethoxy flavone (**12**) which we have found in the stem wood of *T. brownii*. These flavonoids possess one and three methoxy groups, respectively, and thus are suggested to participate in the antifungal effects of the ethyl acetate extracts of the stem wood of *T. brownii*.

## 4. Materials and Methods

### 4.1. Collection of Plant Material

The stem wood and stem bark was collected from many individuals of *Terminalia brownii* growing in natural savannah woodland, in the Blue Nile Forest, in south-eastern Sudan (Figure 1). Voucher specimen were identified by the first author, Mr. Abdelazim Yassin Abdelgadir (Ph.D), Mr. Ashraf Mohamed Ahmed Abd Alla (Ph.D., Wood Sciences) and Mr. Haytham Hashim Gibreel (Ph.D., Taxonomy) at the Faculty of Forestry, University of Khartoum, Sudan and Mr. El Sheikh Abd alla Al Sheikh (Ph.D., Taxonomy) at Soba Forest Research Center, Khartoum, Sudan (Ph.D., Taxonomy). The Voucher specimens are deposited in the herbarium at the Department of Forest Products and Industries, Faculty of Forestry, University of Khartoum, Sudan.

### 4.2. Extraction

Hundred (100) grams of the dried and powdered stem wood and bark were used for the extractions. Extraction was initiated with sequential extraction, beginning with petroleum ether, followed by chloroform and finally the marc was extracted using 80% methanol. The 80% methanolic extract was subjected to liquid/liquid fractionation using ethyl acetate and this fractionation resulted in aqueous and ethyl acetate fractions.

### 4.3. Thin Layer Chromatography (TLC)

Using micro-capillary pipettes, 5 µL of ethyl acetate extracts (5 mg/mL) of the stem bark and wood of *T. brownii* were applied on normal phase silica gel thin layer plates (Kieselgel 60 F254, aluminum backed, Merck, Darmstadt, Germany) and on reversed phase thin layer plates (RP-18 F254s, Merck, Darmstadt, Germany) to detect compounds of a wide range of polarities. Toluene: ethyl acetate: formic acid (4:5:1, *v*:*v*:*v*) was used as an eluent for NP-TLC, while methanol: water: acetic acid (6:2:2) was used for RP-TLC. The development distance was 8 cm. The plates were sprayed with Vanillin-H_2_SO_4_, Dragendorff reagent, aluminum chloride and Natural Products reagents to detect various compound classes such as essential oils, terpenes, phenolic compounds, alkaloids and flavonoids [57]. The plates were observed in UV-light at 254 and 366 nm. A Camaq Video documentation system was used for photographing the plates.

### 4.4. Solid Phase Extraction (SPE)

LC-18 reversed phase cartridges (Supelco, Sigma-Aldrich, Darmstadt, Germany) were used for solid phase extraction in order to purify and enrich flavonoids and for separation of sugars and other interfering matrix compounds. The columns were equilibrated with 100% water and elution was performed using a gradient from 100% to 50% water followed by 100% methanol.

### 4.5. Reversed Phase High Performance Liquid Chromatography Coupled to Diode Array Detection (HPLC-UV/DAD)

The Agilent 1100 series HPLC system was used for the HPLC runs. The system consisted of an Agilent 1100 autosampler connected to Agilent series 1200 binary pump system coupled to an Agilent series 1100 thermostatic column compartment and an Agilent series 1100 DAD detector. Separations were performed on a reversed phase column (Varian LC-18; 4.6 mm × 250 mm; ID 5 µm, USA) at 30 °C and the flow rate was 0.5 mL/min. 5 µL of samples (5 mg/mL in 80% aqueous methanol) were injected. Gradient elution was performed using solvent (A) water +1% of acetic acid to increase peak resolution. Solvent (B) 100% acetonitrile. The step gradient began with 90% A and stopped while reaching 10% B in 30 min. After this 100% B was used for 5 min followed by 10% B for 5 min. Wavelengths of 254, 320, 360 and 380 nm were used for detection. The data was compared to standard compounds and computer libraries of pure compounds.

### 4.6. LC-Triple Quadrupole Mass Spectrometric Analysis (LC-MS and LC-MS/MS Tandem Mass Spectrometry)

An HPLC apparatus (1100 series, Agilent, Waldbronn, Germany) connected to an electrospray ionization (ESI) triple quadruple mass spectrometer (HTC Ultra-Bruker Daltonics-Advanced Mass Spectrometry Instrumentation, Germany) was used. Gradient elution was performed using acetonitrile (MeCN) and water containing 0.005% formic acid (Solvent A) and acetonitrile and glacial acetic acid (Solvent B). A linear gradient from 4% to 33% B was employed for 35 min and was increased to 100% B for 5 min. Then 4% B was used for 5 min to re-equilibrate. Mass analysis of compounds was performed using negative ion mode. The spray voltage was set to 5000 V and the capillary temperature to +280 °C. Nitrogen was used as sheathing gas and the flow was set to 40 U. Collision-induced dissociation (CID-MSn) was applied to induce fragmentation of the molecular ions, and their fragments were analyzed using tandem mass spectrometry. Helium was used as collision gas at 0.8 m Torr. Collision energies of 15 and 30 eV were used to investigate neutral loss and product ions and scanning was performed using a mass range from 50 to 1000 *m*/*z*. Data from the literature, the Wiley Natural product library, and authentic samples were used for the structural identifications of phenolic compounds such as flavonoids, stilbenes and ellagic acid derivatives as well as triterpenes.

### 4.7. Antifungal Assays

#### 4.7.1. Fungal Strains

*Aspergillus niger* ATCC 9763, *Nattrassia mangiferae* ATCC 96293, *Aspergillus flavus* ATCC 9763 and *Fusarium moniliforme* ATCC 24378 were obtained from National Research Center, Sudan. Before use, the strains were sub-cultured on Sabouraud dextrose agar (Oxoid™ CM0041B) slants, at +35 °C.

#### 4.7.2. Agar Well Diffusion Method 

A cup well agar diffusion method [55,56] with minor modifications was used. Before the test, the fungal strains were grown on petri dishes (⌀ = 9 cm) containing Sabouraud dextrose agar at +35 °C overnight [92]. The resulting fungal growth was washed with 100 mL sterile normal saline to obtain fungal suspension containing conidia, which were used for the tests. 200 µL of this fungal suspension was adjusted to 1.0 × 10^8^ CFU/mL and mixed with 20 mL of sterile, molten Sabouraud dextrose agar which was poured into sterile petri dishes (⌀ = 9 cm). The petri dishes were left to set at room temperature. Four holes were cut in the agar using a sterile cork borer (10 mm in diameter) and each hole was filled with 100 µL of extracts (1 mg/mL in 50% methanol) and amphotericin B (Sigma-Aldrich, 1 mg/mL in 50% methanol). 100 µL of 50% methanol was used as a negative control. The extracts/antibiotics/solvents were left to diffuse into the agar in the cold room (+4 °C) for one hour. The plates were then incubated at +35 °C for 24 h. For each experiment four replicates (*n* = 4) were used. The diameters of the zones of inhibition (IZ) were measured in mm using a caliper and the mean of five diameters ± SD and SEM was calculated.

#### 4.7.3. Agar Dilution Method

Minimum inhibitory concentrations were determined using a slightly modified agar macrodilution method [93]. Fungal conidial suspensions were grown for four days in Sabouraud dextrose broth at +35 °C. For the test, 1 mL of these suspensions were diluted with 0.9% (*w*/*v*) NaCl to contain 1.0 × 10^6^ CFU/mL. 100 µL of these fungal suspensions were mixed with 10 mL molten Sabouraud dextrose agar which was pipetted into a petri dish (⌀ = 9 mm). 10 mL of twofold dilutions of plant extracts (from 500 to 31.25 µg/mL) and amphotericin B (from 500 to 15.625 µg/mL) were added to the petri dishes. Each dilution contained 500 µL of 50% methanol or hexane solutions of the plant extracts or antibiotics dissolved in 10 mL of molten Sabouraud dextrose agar. The petri dishes were incubated for 24 h at +35 °C. The MIC was taken as those concentrations that resulted in clear petri dishes showing no visible fungal growth. All tests were performed in triplicates. The solvents used for the plant extractions, 50% methanol or hexane, were used as negative controls. Hexane was used for dissolving those extracts which did not dissolve in 50% MeOH, that is very nonpolar extracts, such as those originating from hexane and petroleum ether extractions.

#### 4.7.4. Statistical Analysis

The Student’s *t*-test provided by Microsoft Excel was used for the evaluation of the statistical significance of any differences between the antifungal results of the inhibition zones (IZ) of the tested extracts.

## 5. Conclusions

Ethyl acetate and aqueous extracts of the stem wood and bark of *Terminalia brownii* give good antifungal effects against *Nattrassia mangiferae*, *Fusarium verticilliodes*, *Aspergillus flavus* and *Aspergillus niger*. Altogether twelve compounds were identified from an ethyl acetate extract of the stem wood of *T. brownii. Cis*- and *trans*-isomers of resveratrol 3-*O*-β-galloyl-glucoside were characterized for the first time in this species of *Terminalia*. Likewise, gallagic acid dilactone has not been reported previously in the stem wood of *T. brownii.* Owing to its relative chemical stability and its reported antifungal efficiency against phytopathogenic molds, gallagic acid dilactone might be an especially interesting component in standardized antifungal extracts of *T. brownii*. Also, standardized extracts of *T. brownii* stem wood, enriched with ellagitannins, could be used as natural fungicides for protecting crops and as medicines to treat fungal infections. Ellagitannins purified from these extracts, if found to be more active than the extracts, could be used for ecological crop plant protection and wood preservation, while being relatively stable and possessing less toxicity than synthetic fungicides.

Our results provide partly the justification for the uses of water-based extracts of *T. brownii* for the protection of crop plants and for wood preservation in Africa, although phytochemical analysis of these aqueous extracts would be needed. Further studies are needed on the antifungal activities of separated compounds from both the aqueous and ethyl acetate extracts as well as on various controlled combinations of these compounds. In summary, standardized extracts of *T. brownii* stem wood could be used as new, cheaper and eco-friendly fungicides for routine use in Africa instead of toxic synthetic fungicides.

## Figures and Tables

**Table 1:** Antifungal activity of stem wood and bark extracts of *T. brownii*. Results were obtained using cup well agar diffusion and agar dilution methods.

Fungal Strain	Stem Wood Extracts		Stem Bark Extracts		Amphotericin-B	
	IZ	MIC	IZ	MIC	IZ	MIC
*Aspergillus niger*						
Pt	NA		NA			
CHCl3	12 ± 0.9		13 ± 0.4			
EtOAc	17± 0.7	500	17 ± 0.8	500	35 ± 0.01	31.25
aqueous	17± 0.5		16.5 ± 0.4			
*Aspergillus flavus*						
Pt	NA		NA			
CHCl3	14 ± 0.5		14 ± 0.9			
EtOAc	18.5 ± 0.4	500	18.5 ± 0.8	500	28 ± 0.03	125
aqueous	18 ± 0.9		18 ± 0.5			
*Nattrassia mangiferae*						
Pt	NA		NA			
CHCl3	12 ± 0.5		12± 0.7			
EtOAc	19 ± 0.4	250	18.5 ± 0.4	250	30 ± 0.04	62.5
aqueous	18.5 ± 0.4		19 ± 0.4			
*Fusarium verticillioides*						
Pt	NA		NA			
CHCl3	13 ± 0.6		11 ± 0.9			
EtOAc	20 ± 0.4	250	19 ± 0.2	250	31 ± 0.03	62.5
aqueous	19 ± 0.3		18 ± 0.7			

For agar diffusion, extracts at the concentration 1 mg/mL were used. Diameter of inhibition zones (IZ) in mm: >18 mm: sensitive; 14–18 mm: intermediate; <14 mm: resistant [55,56]; Pt, petroleum ether extracts; CHCL3, chloroform extracts; EtOAc, ethyl acetate extracts; NA, Not active. IZ results as mean ± SEM of five measurements. MIC in µg/mL. The observed differences between the sample means of the inhibition zones (the stem bark and wood extracts) against the tested fungi did not differ significantly.

**Table 2:** HPLC-DAD and MS/MS data of phenolic compounds and triterpenoids in an ethyl acetate extract of the stem wood of *T. brownii*.

Peak No	Rt (min)	[M-H] *(m/z)*	CID M^n^ Main Fragment Ions *(m/z)*	Identified Compound	Molecular Formula	Exact Mass (Calc.)
1	6.8	469	425, 407, 379, 353, 300, 271	oleanane type triterpenoid	-	-
2	6.8	491	447, 429, 411, 401, 385, 301	oleanane type triterpenoid	-	-
3	11.1	541	532, 425, 397, 301, 273, 227, 199, 169	*cis*-resveratrol-3-*O*-β-galloyl-glucoside	C_27_H_26_O_12_	542.1416
4	13.2	541	532, 424, 407, 300, 275, 227, 199, 169	*trans*-resveratrol-3-*O*-β-galloyl-glucoside	C_27_H_26_O_12_	542.1416
5	14.1	483	451, 433, 407, 305, 405, 377	Methyl-(*S*)-flavogallonate	C_22_H_12_O_13_	484.0273
6	14.4	601	583, 301, 299, 271, 243, 215	Gallagic acid dilactone	C_28_H_10_O_16_	601.9964
7	15.3	433	300, 314, 229, 271, 132	Naringenin-4′-methoxy-7-pyranoside	-	-
8	16.8	625	301, 284, 256, 229, 201,185, 129	Quercetin-7-β-*O*-diglucoside	C_27_H_30_O_17_	626.1473
9	18.2	633	481, 463, 421, 387, 305, 275, 300, 169	Corilagin derivative	-	-
10	18.4	585	301, 284, 257, 229, 201, 185, 153, 132	Quercetin-7-*O*-galloyl-glucoside	-	-
11	19.1	725	665, 503, 409, 441, 379, 391	Unknown ellagitannin	-	-
12	25.5	343	328, 313, 298, 285, 270, 257	5,6-dihydroxy-3′,4′,7-trimethoxy-flavone	-	-

Rt, retention time in HPLC-DAD; [M-H ]^−^ *(m/z)*, base or molecular ions at negative mode; CID M^n^, Fragmentation ions resulting from collision-induced dissociation; The exact mass (calc.) according to the molecular formula of identified compounds. Aglycones are underlined. Peak numbers according to Figure 3.

## Supplementary Materials

The following are available online at www.mdpi.com/2079-6382/6/4/37/s1, (Slides 1–12): Tandem mass spectra of molecular ions and their fragment product ions resulting from selected compounds in a *T. brownii* ethyl acetate extract of the wood.
