# Supplementary figures and images for: LC-MS/MS Tandem Mass Spectrometry for Analysis of Phenolic Compounds and Pentacyclic Triterpenes in Antifungal Extracts of Terminalia brownii (Fresen)
Source: Antibiotics (Basel). 2017 Dec 13;6(4):37. doi: 10.3390/antibiotics6040037 (PMC5745480; doi:10.3390/antibiotics6040037)

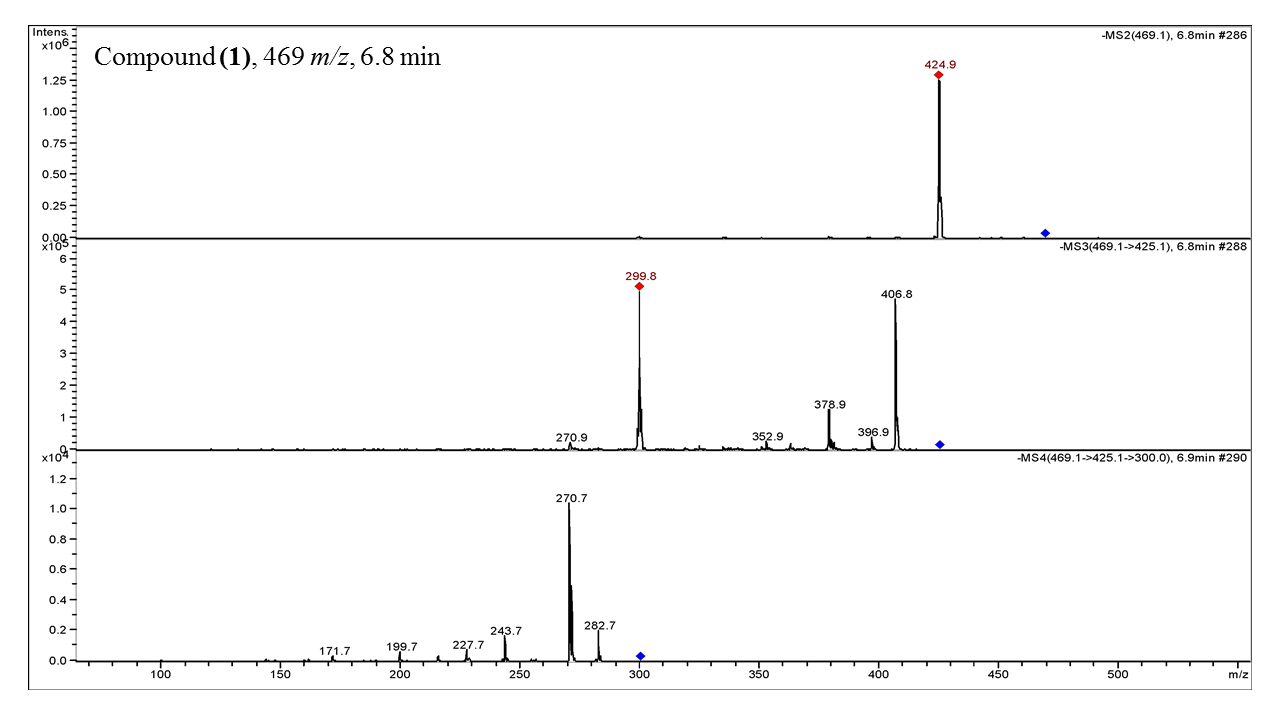

Supplement: Supplementary file 1 [file antibiotics-06-00037-s001.zip › Supplementary Materials/Slide1.TIF]

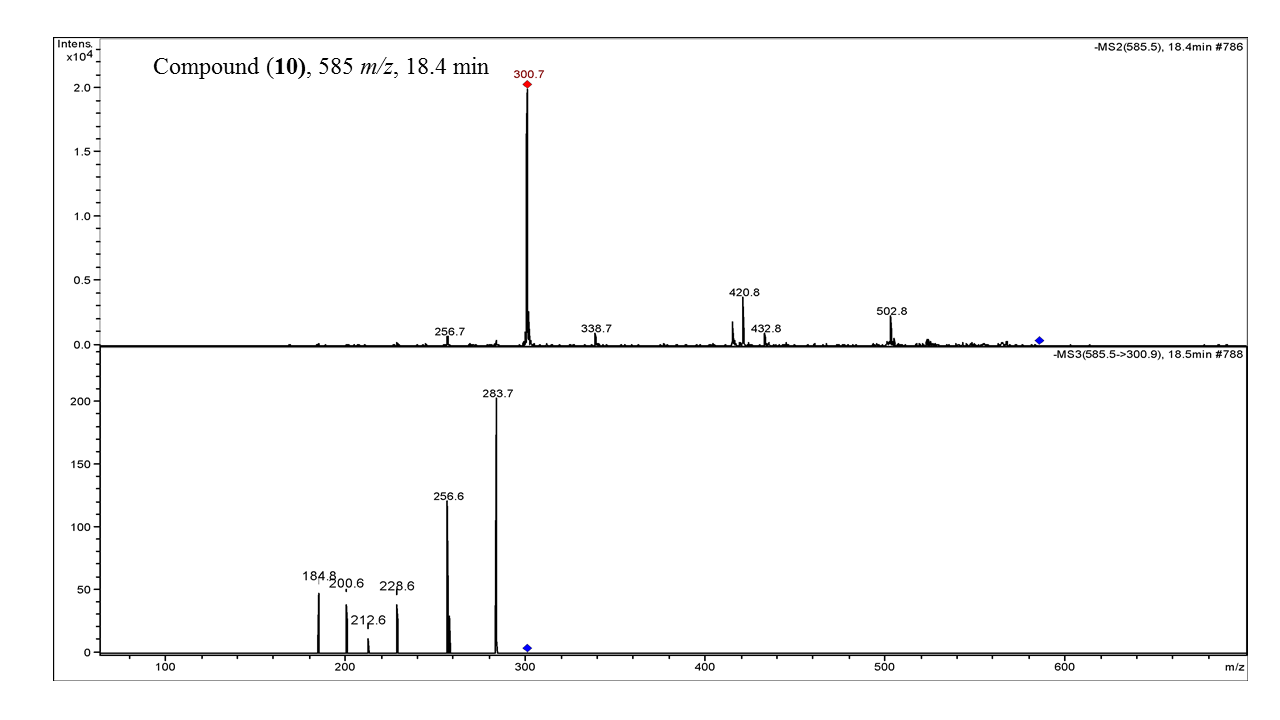

Supplement: Supplementary file 1 [file antibiotics-06-00037-s001.zip › Supplementary Materials/Slide10.TIF]

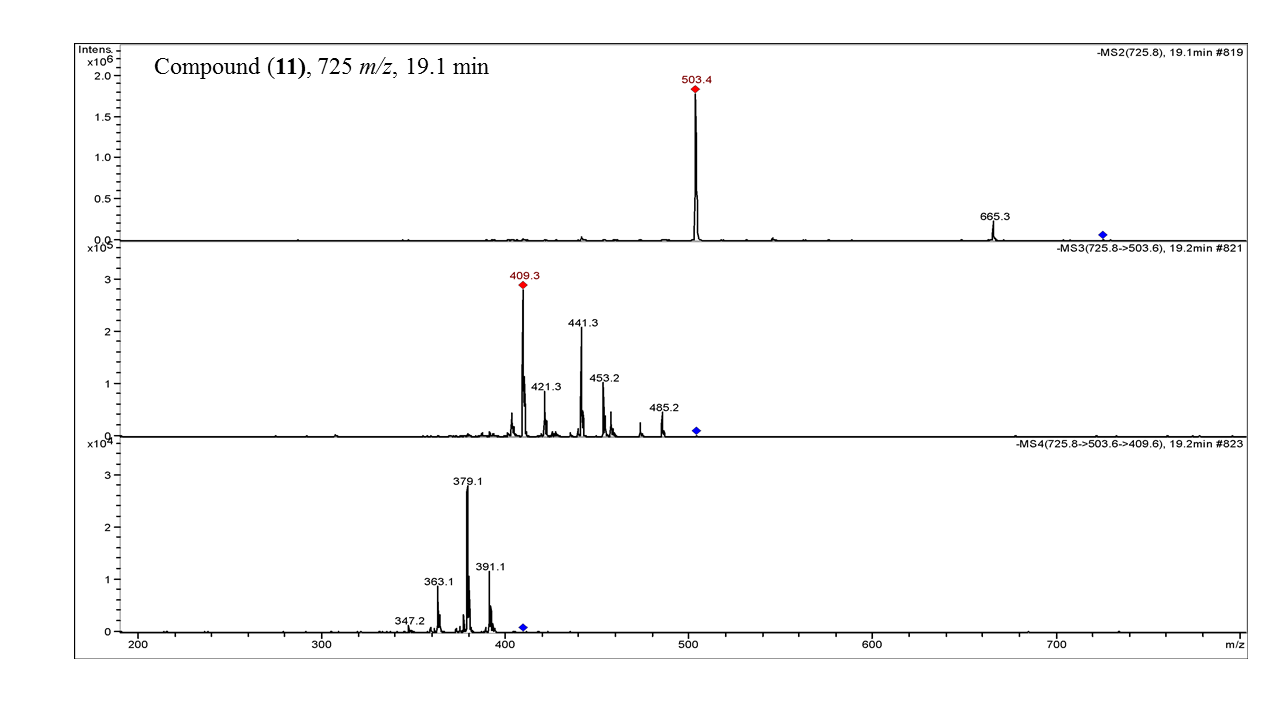

Supplement: Supplementary file 1 [file antibiotics-06-00037-s001.zip › Supplementary Materials/Slide11.TIF]

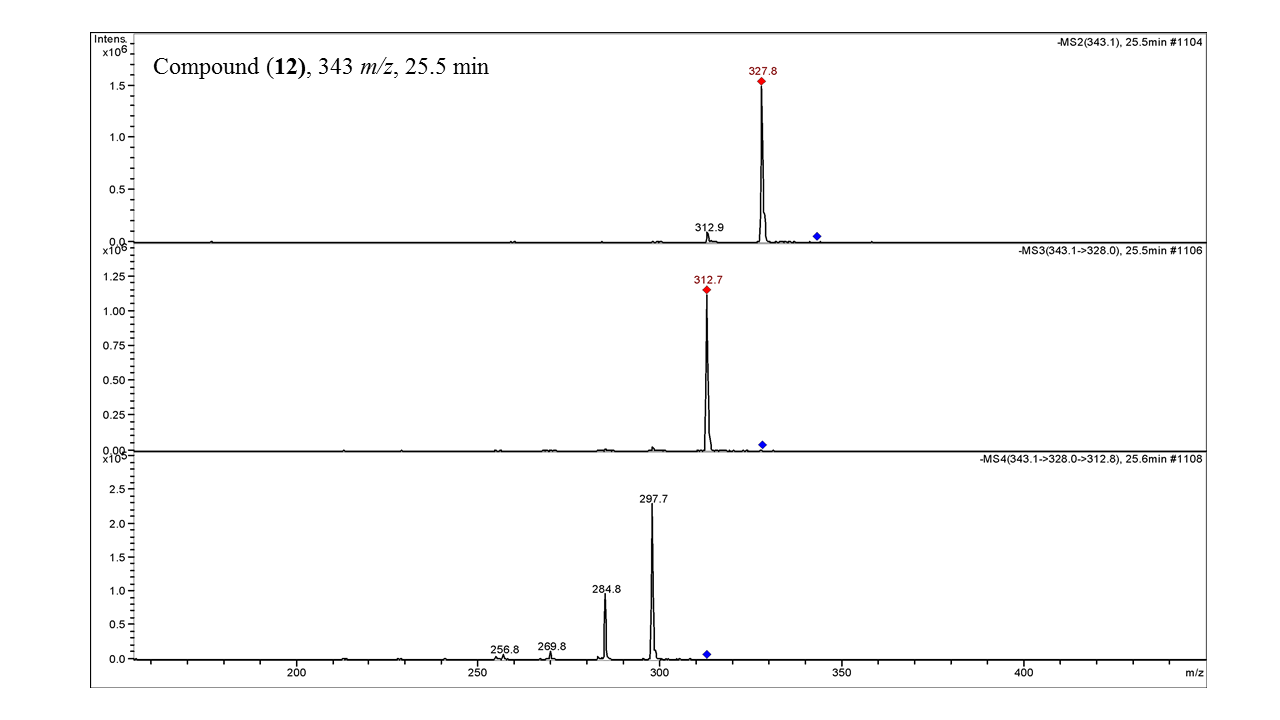

Supplement: Supplementary file 1 [file antibiotics-06-00037-s001.zip › Supplementary Materials/Slide12.TIF]

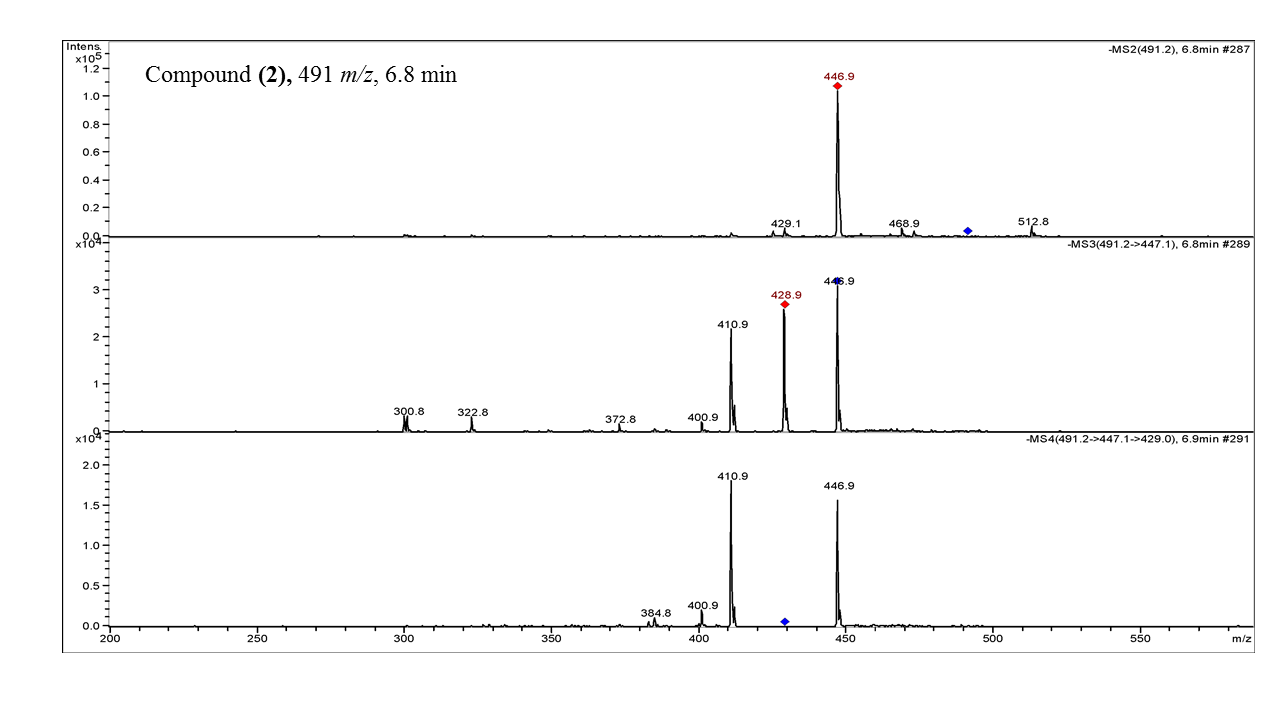

Supplement: Supplementary file 1 [file antibiotics-06-00037-s001.zip › Supplementary Materials/Slide2.TIF]

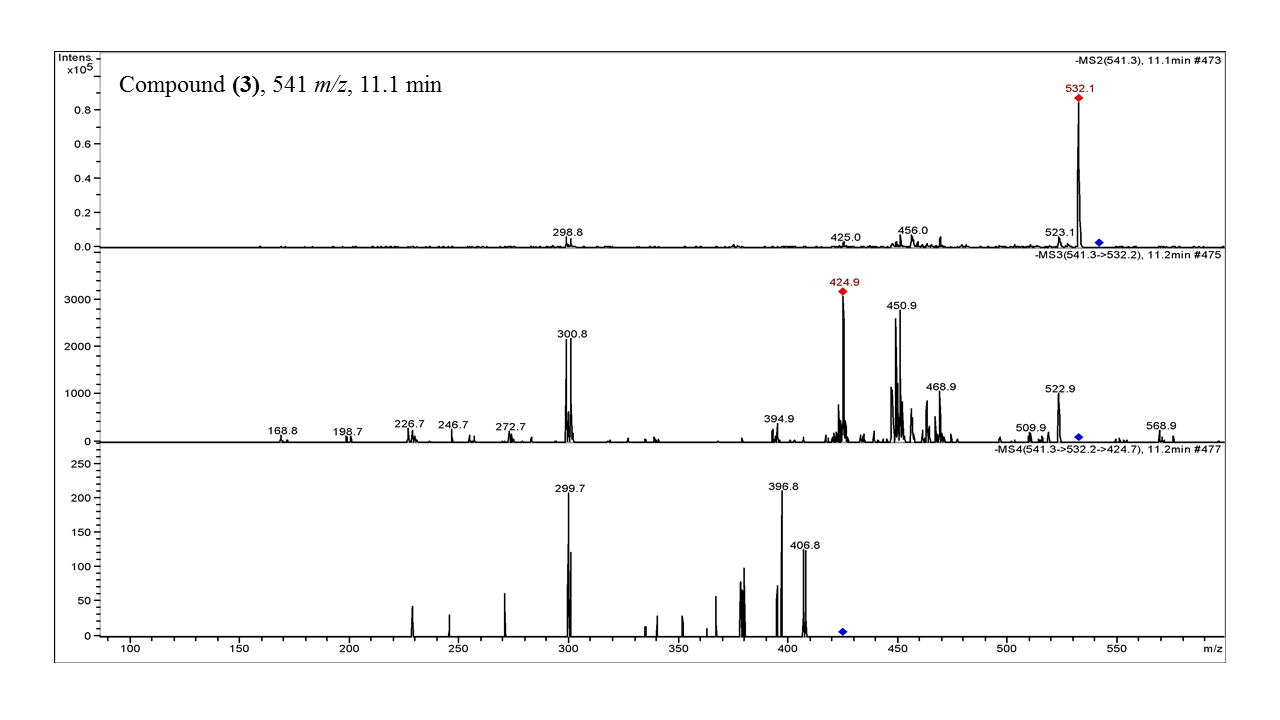

Supplement: Supplementary file 1 [file antibiotics-06-00037-s001.zip › Supplementary Materials/Slide3.TIF]

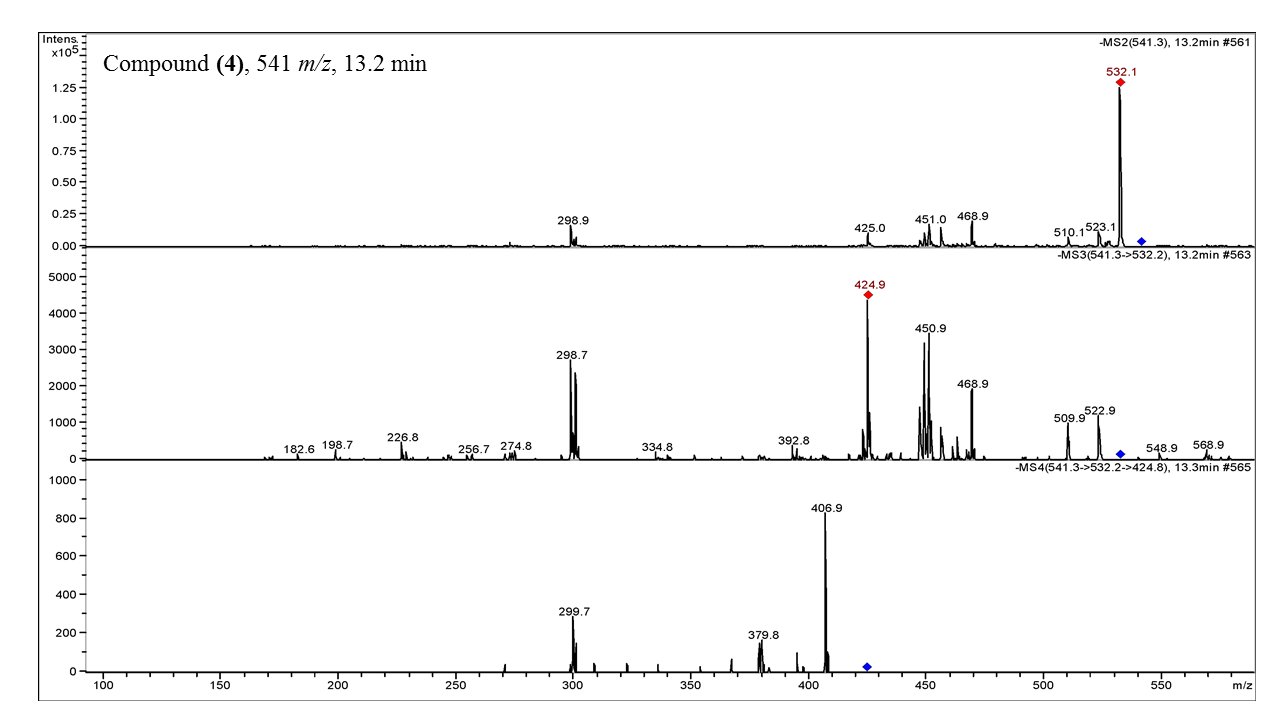

Supplement: Supplementary file 1 [file antibiotics-06-00037-s001.zip › Supplementary Materials/Slide4.TIF]

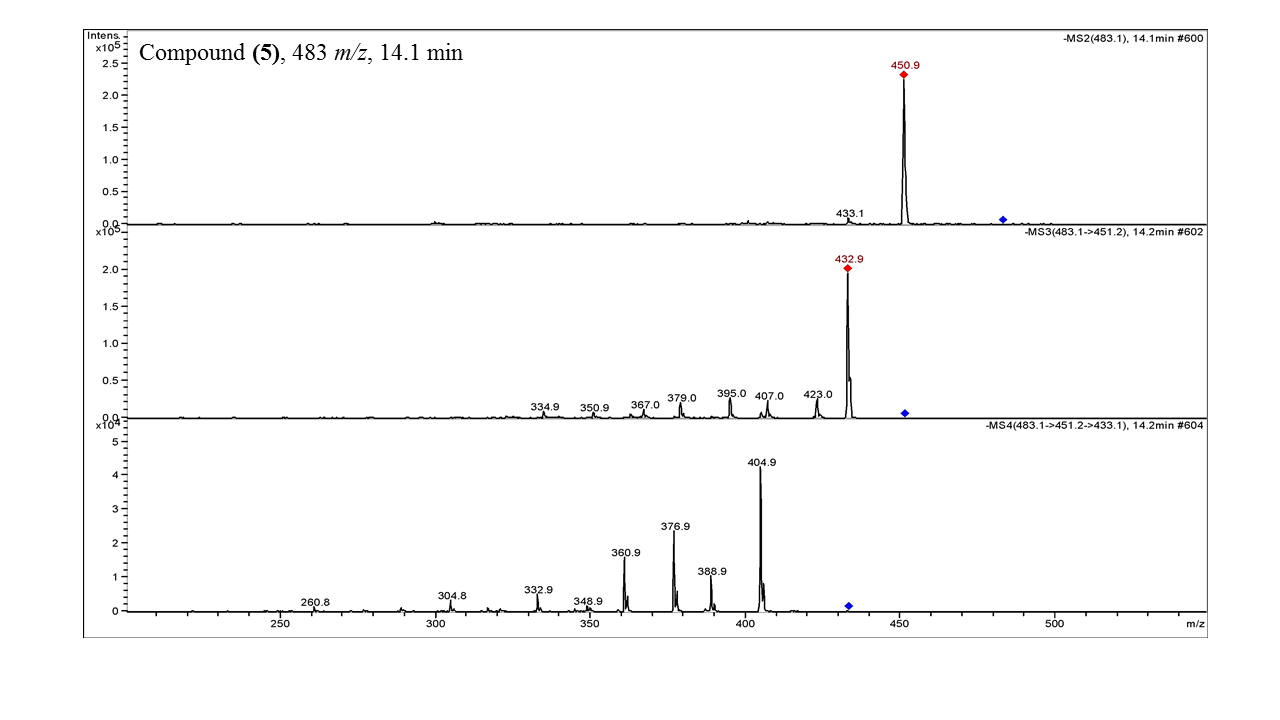

Supplement: Supplementary file 1 [file antibiotics-06-00037-s001.zip › Supplementary Materials/Slide5.TIF]

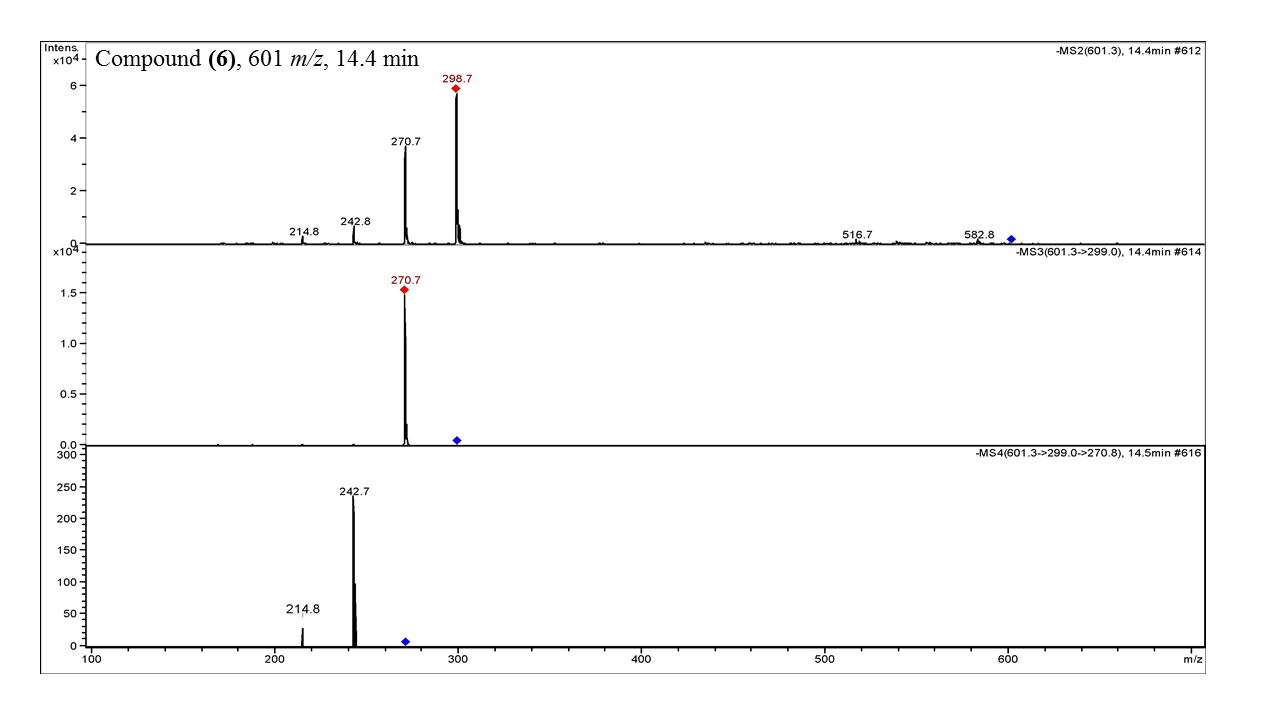

Supplement: Supplementary file 1 [file antibiotics-06-00037-s001.zip › Supplementary Materials/Slide6.TIF]

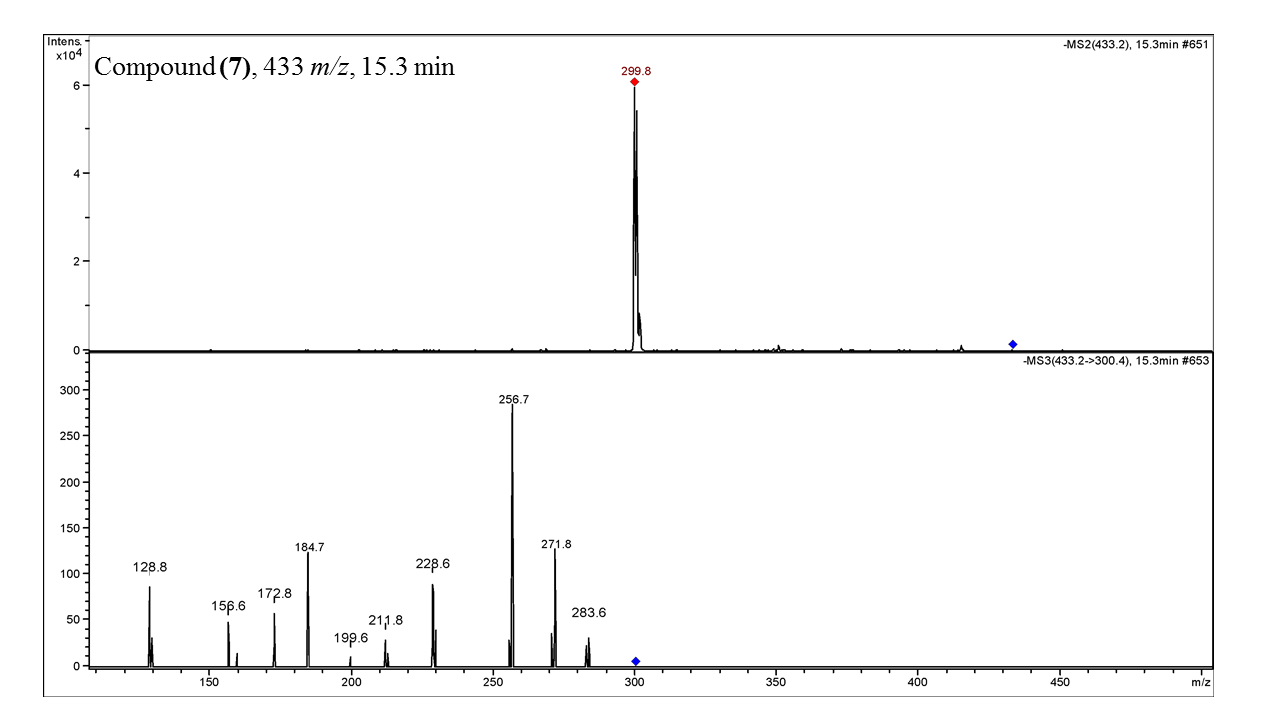

Supplement: Supplementary file 1 [file antibiotics-06-00037-s001.zip › Supplementary Materials/Slide7.TIF]

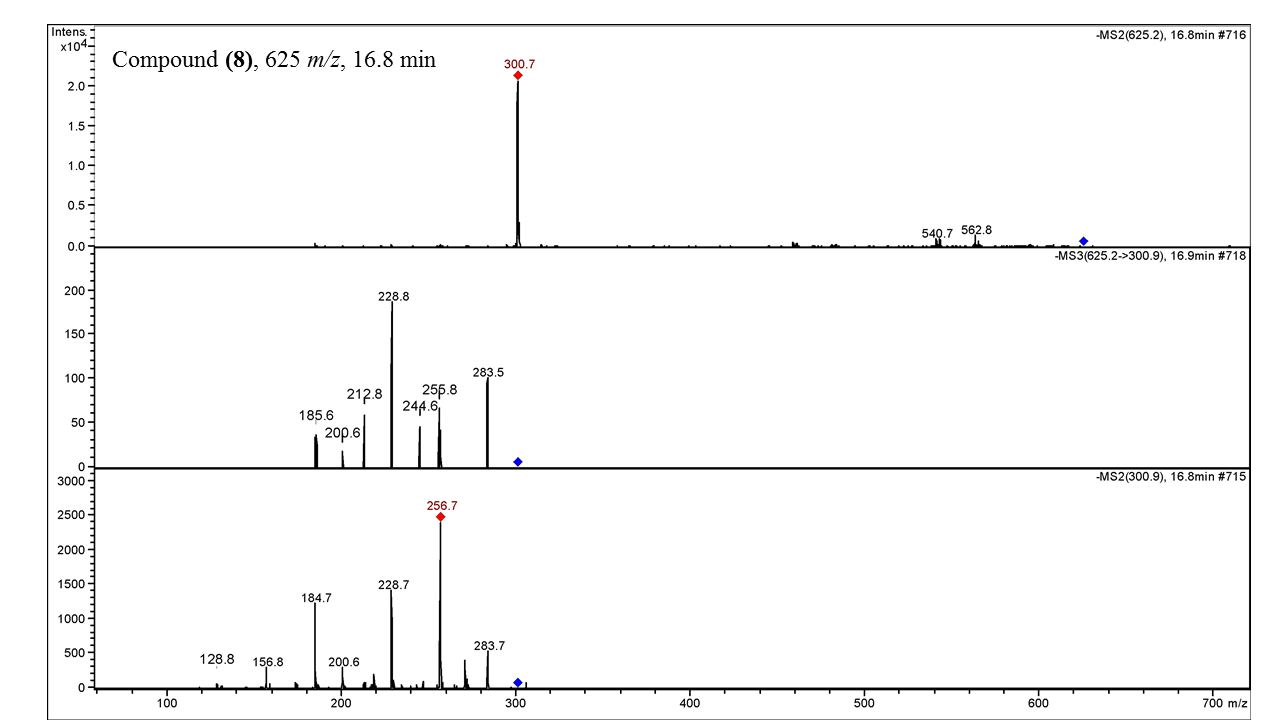

Supplement: Supplementary file 1 [file antibiotics-06-00037-s001.zip › Supplementary Materials/Slide8.TIF]

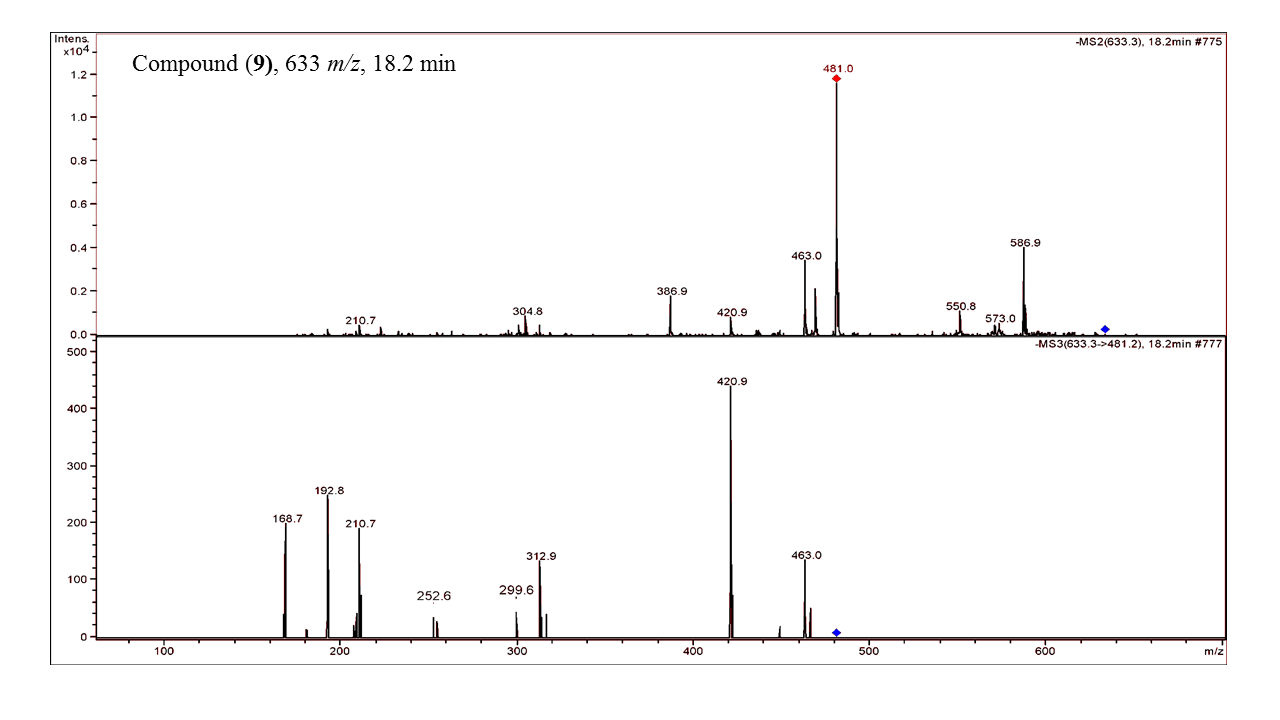

Supplement: Supplementary file 1 [file antibiotics-06-00037-s001.zip › Supplementary Materials/Slide9.TIF]
